# Next‐Generation Manufacturing: The Evolving Role of Biocatalysis in AstraZeneca

**DOI:** 10.1002/cbic.70489

**Published:** 2026-09-14

**Authors:** Alba Diaz‐Rodriguez, Martin A. Hayes, Keith R. Mulholland, Fernando Tur, Samantha Staniland

**Affiliations:** ^1^ Early Chemical Development Pharmaceutical Sciences Biopharmaceuticals R&D AstraZeneca Cambridge UK; ^2^ Discovery Sciences Biopharmaceuticals R&D AstraZeneca Gothenburg Sweden; ^3^ School of Chemistry and Molecular Biosciences The University of Queensland St Lucia Australia; ^4^ Chemical Development Process Technology and Development Operations AstraZeneca Macclesfield UK; ^5^ Early Chemical Development Pharmaceutical Sciences Biopharmaceuticals R&D AstraZeneca Macclesfield UK; ^6^ Oncology R&D AstraZeneca Cambridge UK

**Keywords:** biocatalysis, enzyme engineering, green chemistry, process development, sustainability

## Abstract

Biocatalysis is becoming a strategic manufacturing capability at AstraZeneca, enabling selective transformations under milder conditions and with the potential to reduce waste and resource intensity relative to conventional routes. This review summarizes how AstraZeneca is embedding biocatalysis across the portfolio to support its long‐term net‐zero commitments. We describe capability building across people, through expansion of cross‐site expertise, automated high‐throughput experimentation platforms, and academic partnerships that extend innovation and capacity. Case studies show how enzyme identification, optimization, and directed evolution translate into robust, scalable processes for active pharmaceutical ingredients. We close by defining priorities—end‐to‐end automated workflows and artificial intelligence‐enabled design—to shorten enzyme engineering cycles and broaden biocatalysis impact across modalities.

## Graphical abstract

Biocatalysis is strengthening AstraZeneca's manufacture of active pharmaceutical ingredients through faster development, robust scale‐up, and lower environmental impact. Case studies illustrate where biocatalysis delivers greatest value, while enabling platforms, teams, and partnerships shape its next phase.

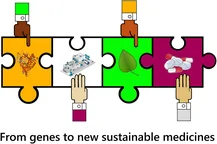

## 1 Introduction

The pharmaceutical industry faces the urgent challenge of delivering effective medicines while significantly reducing its environmental impact [1].

AstraZeneca has responded with its Ambition Zero Carbon initiative, pledging to achieve science‐based, net‐zero greenhouse gas emissions across all operations and its entire value chain by 2045 [2]. Manufacturing activities constitute a major share of AstraZeneca's overall greenhouse gas emissions, with value‐chain–associated emissions (including purchased materials, transport and product use) exceeding six million tons of CO_2_‐equivalent annually [2]. Because these emissions arise predominantly from large‐scale production and upstream supply chains, decisions made during early R&D exert a substantial influence on the eventual environmental footprint of commercial manufacture. Integrating sustainability considerations into chemical process development is therefore essential to reducing the carbon intensity of pharmaceutical production and enabling progress toward AstraZeneca's net‐zero commitments.

A core component of AstraZeneca's approach is the integration of green chemistry, which emphasizes the design of chemical processes that maximize resource efficiency, minimize hazardous substances, and reduce waste [3, 4, 5]. These principles guide decision‐making throughout drug development, influencing areas such as the selection of safer reagents, reducing process mass intensity, and lowering energy consumption [6, 7, 8]. These concepts are applied across development, scale‐up, and manufacturing, supported by ongoing efforts in process intensification, solvent minimization, and use of alternative feedstocks [9, 10, 11].

Within this framework, biocatalysis [12, 13, 14, 15, 16] is a transformative technology that provides solutions for the increasing complexity of pharmaceuticals [17, 18, 19]. Over the past 2 decades, biocatalysis has evolved from a niche‐enabling technology to an increasingly established component of pharmaceutical process development and manufacturing, with numerous examples of successful implementation across the industry [20]. Enzymes enable key transformations with exquisite stereocontrol under mild conditions, often reducing both carbon and resource intensity compared to traditional chemical methods [21, 22]. Advances in genomics [23, 24, 25], protein engineering [26, 27, 28], machine learning [29, 30], and high‐throughput experimentation (HTE) [31, 32, 33, 34, 35] have expanded the toolbox of available biocatalysts, making it possible to discover and optimize enzymes for non‐natural substrates [36, 37].

While the scientific foundations and industrial track record of biocatalysis continue to strengthen, its broader implementation remains constrained by practical challenges associated with speed, predictability, and integration into pharmaceutical development workflows. Translating sustainability ambitions across multiple fast‐paced drug‐development programs is challenging because key route decisions are made under compressed timelines, limited information about the eventual candidate, and frequent changes in priorities [17]. In this context, teams often default to established chemistries because they are perceived as lower risk for rapid delivery. Biocatalysis can be deprioritized not because it lacks scientific promise, but because identifying a suitable enzyme and subsequently improving its activity, selectivity, and robustness often requires iterative screening and engineering cycles that may not align with discovery‐stage timelines, resourcing, or decision windows.

The central barrier is therefore one of delivery: reducing uncertainty and time‐to‐decision so that enzymatic solutions can be evaluated and progressed with the same confidence as conventional chemistry. Addressing these barriers requires not only advances in enzyme technology but also the organizational capabilities needed to deploy biocatalysis efficiently across diverse projects. Accordingly, this review frames AstraZeneca's response around three levers—people, platforms, and partnerships—that collectively accelerate the implementation of biocatalytic steps into scalable manufacture.

## 2 Capability Building

AstraZeneca has advanced biocatalysis from a specialist option to a strategic capability for delivering more efficient and often more sustainable chemical routes. As more synthetic options must be evaluated earlier—when time and expert capacity are constrained—success depends on foundations that enable fast, confident decisions. Two needs dominate industrial deployment: easy access to diverse enzymes and rapid screening and optimization to close performance gaps. At AstraZeneca, these are delivered through cross‐site expertise, high‐throughput experimentation platforms, and partnerships that expand enzyme access and capacity. Together, they improve starting points, shorten optimization cycles, and bring biocatalytic steps to process readiness earlier, reducing late‐stage risk and enabling more routes to progress in parallel.

### 2.1 People

AstraZeneca's biocatalysis capability was established well before 2020 but was initially delivered by a small team supporting individual projects. Sustained investment in people—through targeted recruitment and upskilling—has since expanded the routine use of enzymatic methods across the portfolio. A key step was the establishment in 2020 of a dedicated group at the Cambridge R&D site (UK) to build in‐house directed evolution, enabling systematic improvement of enzyme performance when project requirements demand it. This has grown into a cross‐site community of ~20 scientists across three locations, spanning enzyme engineering through process development and implementation in both early‐ and late‐stage programs.

### 2.2 Platforms

Historically, the routine use of biocatalysis was limited by the slow and labor‐intensive nature of traditional screening workflows, which made enzyme evaluation difficult to align with fast project timelines. To overcome these constraints, AstraZeneca has invested in high‐throughput experimentation and automation platforms, enabling rapid assay execution, parallel screening, and accelerated data processing.

**FIGURE 1:**
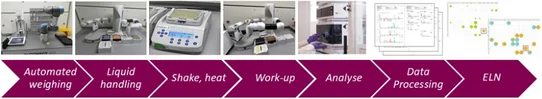
Investment in high‐throughput experimentation for enzyme screening workflows.

Since 2022, automated liquid handling, 96‐well enzyme panels and plate‐based analytical methods have increased screening throughput and shortened the time required to identify viable enzymatic disconnections (Figure 1). This infrastructure supports earlier and more consistent evaluation of enzyme options across projects for rapid decision‐making.

**FIGURE 2:**
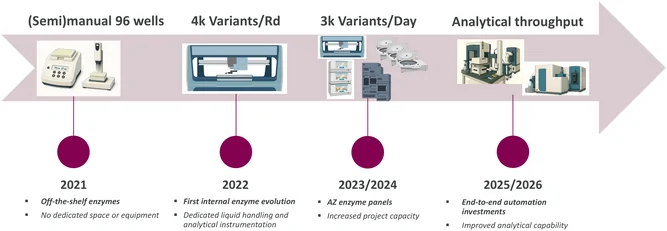
Expansion of directed‐evolution screening capacity.

In 2023, AstraZeneca launched a multi‐year program to strengthen internal biocatalysis capabilities spanning high‐throughput screening, directed evolution, and process development. Phase 1 established core infrastructure and expanded proprietary enzyme panels, enabling faster iteration and retention of enzyme variants and associated know‐how. Current Phase 2 is expanding capacity across multiple sites and improving delivery through targeted automation and enhanced data workflows. Together, these investments will enable faster iteration cycles, closer alignment with process needs, and broader adoption of biocatalysis across the organization (Figure 2).

**FIGURE 3:**
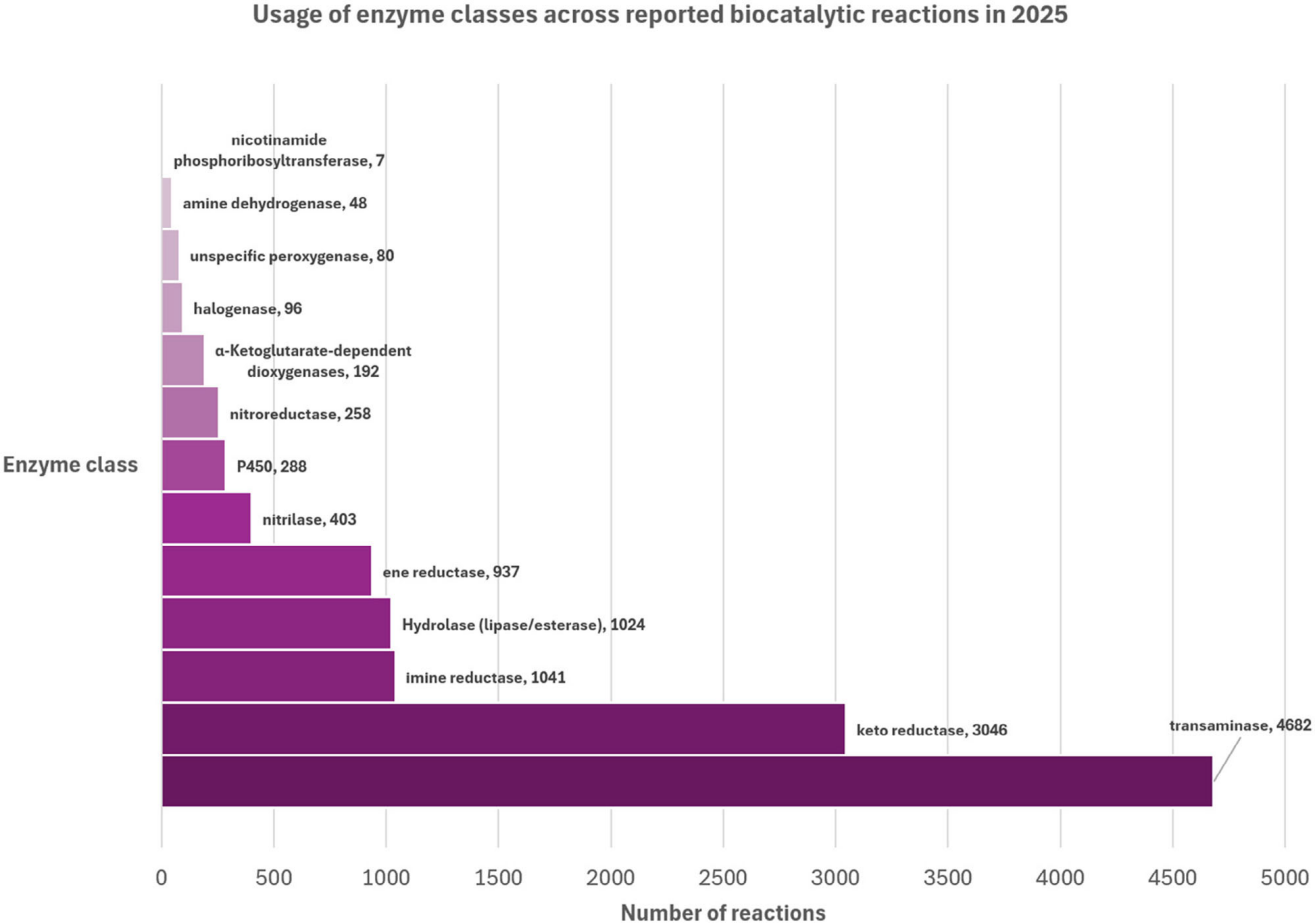
Most‐used enzyme classes by number of internal reactions in 2025 (excluding enzyme evolution projects).

The expanded capacity has enabled the assessment of biocatalytic options across approximately 25 projects, reflecting both increased demand and the ability to support multiple programs in parallel. Through reported biocatalytic reactions (excluding enzyme engineering projects) in the 2025 dataset (Figure 3), enzyme use is strongly skewed toward a small number of workhorse classes. Transaminases (ATA) (4,682 reactions) and alcohol dehydrogenases (ADH) commonly referred to as ketone reductases (KRED) when used in the reductive direction (3,046) dominate, together accounting for ~64% of all reactions. A second tier comprises imine reductases (IRED) (1,041), hydrolases (lipases/esterases) (1,024), and ene reductases (ERED) (937), after which reaction counts drop sharply, with remaining classes each contributing <5% individually (for example, nitrilases 403; P450s 288). Overall, the distribution highlights a mature core toolkit underpinning most reported transformations, alongside a long tail of less frequently deployed enzyme classes. These data align with trends across the pharmaceutical sector, where these families consistently rank among the most widely used and best‐performing biocatalysts. This concordance reflects their maturity, predictability, and the breadth of applicable transformations [19].

Capability building in screening and analytics has accelerated enzyme evaluation, enabling broader panels to be tested earlier and in parallel across projects and making enzymatic feasibility a front‐loaded decision in retrosynthesis. To institutionalize this shift, enzymatic disconnections are embedded in AstraZeneca's Route Manager, enabling like‐for‐like comparison with conventional chemistry using shared metrics (for example, cost of goods and process mass intensity). Together, these platforms move biocatalysis from opportunistic use to a routine, strategic option and trigger earlier optimization when warranted. Ultimately, this approach increases the likelihood that the most efficient and sustainable route is selected from the outset.

### 2.3 Academic–Industry Partnerships

Academic–industry partnerships accelerate biocatalysis by linking fundamental innovation with rapid translational development [38].

AstraZeneca has a long‐standing record of strategic collaborations that extend its internal capabilities in this space. These partnerships form a critical third pillar of the company's biocatalysis strategy, enabling access to emerging methodologies and supporting the development of solutions for future portfolio needs.

Through these alliances, the organization can access complementary scientific expertise—including novel enzymes, mechanistic insight, and advanced engineering approaches—while directing research toward problems of clear industrial relevance. They also strengthen the talent pipeline by training the next generation of biocatalysis specialists. The collaborations and their contributions to biocatalysis capability building and translation are listed in Table 1.

**TABLE 1:** Selected AstraZeneca academic–industry partnerships supporting biocatalysis capability building and translation.

Period/status	Partnership/program	Partner/collaborator/mentor	Main biocatalysis focus	Key outcome/contribution
2005–2025	CoEBio3: Centre of Excellence in Biocatalysis, Biotransformations and Biocatalytic Manufacture	Manchester Institute of Biotechnology and partner universities; led by Prof. Nicholas Turner	Broad biocatalysis training, discovery, and translational research	Long‐standing talent pipeline and access to world‐class academic expertise across Manchester, York, Liverpool, and Bristol; AstraZeneca supported every CoEBio3 cohort from Stage I to Stage V [39] helping establish one of the UK's most influential biocatalysis training and translation programs.
2007–present	University of Queensland/AZ/ARC‐supported collaborations	University of Queensland, Prof. Elizabeth Gillam, Prof. James de Voss; with MIB involvement	Human and bacterial P450 engineering	Applied ancestral sequence reconstruction to generate thermostable P450 enzymes with improved expression, solvent tolerance, and operational robustness [40].
Over the last decade	AstraZeneca Postdoc Programme [41]	Leading academic groups worldwide including Prof. Eric Carreira, ETH; Prof. Gideon Grogan, York; Prof. Todd Hyster, Princeton; Prof. Bernhard Hauer, Stuttgart; and Prof. Uwe Bornscheuer, Greifswald	Cutting‐edge biocatalysis projects aligned with industrial challenges	Supported ~120 postdoctoral researchers across AstraZeneca at any given time; in biocatalysis delivered projects on epoxide hydrolases [42], UPO‐catalyzed oxidations [43], ERED‐catalyzed C–C coupling [44], and methyltransferase engineering [45].
2019–2024	UKRI Prosperity Partnership/Centre for Biocatalytic Synthesis of New Modalities (CBNM)	Manchester Institute of Biotechnology and Prozomix Ltd; PIs Prof. Nicholas Turner, Prof. Sabine Flitsch, Prof. Perdita Barran, Prof. Nigel Scrutton	Biocatalytic synthesis of new pharmaceutical modalities	Established the Centre for Biocatalytic Synthesis of New Modalities and delivered advances in peptide/protein modification, nucleoside synthesis, and enzymatic amide formation, enabling analytical technologies [46].
2019–present	University of Bristol collaboration with BBSRC support	University of Bristol	Diels–Alderases and enzyme library development	Generated a comprehensive Diels–Alderase library and enabled the first use of a Diels–Alderase in total synthesis of (–)‐13‐deoxytetrodecamycin.
2022–2030	MISTRA SafeChem	KTH Stockholm, Prof. P. O. Syrén and collaborators	Safer and more sustainable biocatalytic amide bond formation	Focus on McbA and ancestral‐sequence‐reconstruction‐derived variants for biocatalytic amide synthesis.
Ongoing	Horizon Europe and UK collaborative networks	PharmECO, BiodeCCodiNNg, HaloVerse, CirculH2, ELEGANCE, NEXTMARINE, and UK C Loop Hub	Sustainable manufacturing, enzyme discovery, AI‐guided biocatalysis, and emerging enzyme chemistry	Enables shared learning, complementary expertise, and coordinated biocatalysis innovation across academia and industry.
Ongoing	AstraZeneca BioVentureHub and BridgeHouse	SMEs and external research groups co‐located at AstraZeneca Gothenburg	Green chemistry, biocatalysis, and sustainability technologies	Enables close interaction with external innovators, access to infrastructure, and coordinated project delivery toward practical application.
From 2026	Industrial Doctoral Landscape Award transition	CoEBio3 successor model; industry and UKRI; led by Prof. Anthony Green at MIB	Doctoral training and industrial biocatalysis research	Continues the CoEBio3 model as an industry–UKRI‐funded doctoral training platform for future biocatalysis talent and innovation [47].

Selected examples are highlighted below to illustrate key advances enabled through these partnerships.

Significant progress has been achieved in site‐selective modification of peptides, proteins, and antibodies. One strategy enables high drug‐to‐antibody ratios (DAR) on native antibodies through selective galactosylation followed by galactose oxidase (GOase) oxidation, generating aldehydes suitable for functionalization via a bioorthogonal tandem Knoevenagel–Michael sequence [48].

Complementary work has shown that Horner–Wadsworth–Emmons (HWE) olefination provides a robust, chemoselective method to modify aldehyde‐containing peptides and proteins under physiologically compatible conditions. This approach has been applied to therapeutic antibodies and stable recombinant proteins, demonstrating broad scope and operational simplicity [49].

A highly selective N‐terminal modification strategy has been developed by exploiting ATP‐dependent formation of acyl‐adenosine monophosphates using the adenylation domain of carboxylic acid reductase from *Segniliparus rugosus* (CARsr‐A). This method has been applied to clinically relevant peptides including liraglutide, glucagon, and insulin [50].

**SCHEME 1:**
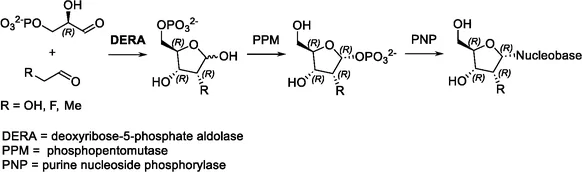
Multienzyme pathway enabling 2′‐stereocontrol and functional diversification in nucleoside synthesis.

Advances have also been made in biocatalytic synthesis of 2′‐modified nucleosides, key intermediates for oligonucleotide therapeutics. Willmot and co‐workers engineered deoxyribose‐5‐phosphate aldolase (DERA) mutants capable of producing D‐ribose‐5‐phosphate analogs with strict stereocontrol, enabling promising routes to 2′‐modified purines [51] (Scheme 1).

**SCHEME 2:**
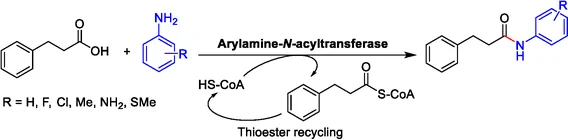
Enzymatic recycling of CoA‐thioesters to support one‐pot acyl‐*S*‐CoA‐dependent transformations.

Given the central role of amide bond formation in drug synthesis, sustainable enzymatic approaches remain a priority. Schnepel and colleagues showed that the adenylation domain of a carboxylic acid reductase (CARsr‐A) can generate coenzyme A (CoA) thioesters from a wide range of carboxylic acids and—when coupled with *N*‐acyl transferases in a one‐pot cascade—enable efficient synthesis of structurally diverse amides (Scheme 2) [52].

Complementing this, AstraZeneca's partnership in the Swedish Miljöinstitutet‐funded MISTRA SafeChem program [53] also focuses on biocatalytic amide bond formation using the ATP‐dependent amide bond synthetase (ABS) enzyme McbA and variants generated through ancestral sequence reconstruction [54, 55, 56].

Additional collaborations have advanced enabling technologies that support biocatalyst discovery and application with focus on late‐stage modifications [57]. These include the engineering and deployment of reductive aminases (RedAms) for efficient stereoselective reductive amination [58]; new mechanistic insights into flavin‐dependent halogenases [59], clarifying determinants of regio‐ and chemoselective halogenation; and substantial improvements in high‐throughput analytical methodologies, notably enhanced desorption electrospray ionization mass spectrometry workflows that significantly accelerate screening and reaction characterization [60].

**SCHEME 3:**
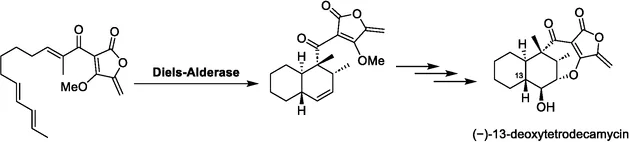
Enantioselective Diels–Alderase–mediated cascade toward (–)‐13‐ deoxytetrodecamycin.

The collaboration with the University of Bristol on Diels–Alderases resulted in a comprehensive enzyme library and the first application of a Diels–Alderase in a total synthesis—(–)‐13‐deoxytetrodecamycin—marking a transition from academic curiosity to practical synthetic utility (Scheme 3) [61, 62].

Beyond these specific academic partnerships, broader collaborative networks have also contributed substantially to the advancement of biocatalysis at AstraZeneca.

Specifically, the organization contributes to Horizon Europe networks including PharmECO (safe‐and‐sustainable‐by‐design approaches and sustainability assessment in pharmaceutical manufacturing) [63], BiodeCCodiNNg (expanding enzymatic reaction chemistry) [64], HaloVerse (enzymatic halogenation) [65], CirculH2 (hydrogenase‐enabled H_2_ biocatalysis) [66], ELEGANCE (data/AI‐guided enzyme and bioprocess design) [67], and NEXTMARINE (mining extreme marine ecosystems for new enzymes) [68], alongside the UK Carbon‐Loop Sustainable Biomanufacturing Hub (C‐Loop) focused on engineering biology routes that valorize carbon‐rich waste streams [69]. These networks provide shared learning opportunities and help align innovation across industry and academia.

Alongside its involvement in European and UK‐based consortia, AstraZeneca also drives collaboration through the AstraZeneca BioVentureHub. The BioVentureHub [70] integrates external small‐ and medium‐sized enterprises (SMEs) and research groups into the Gothenburg R&D site, providing access to laboratory infrastructure, technologies, and scientific expertise. Co‐location with AstraZeneca teams promotes close scientific interaction and helps SMEs accelerate the translation of products and services toward practical application. The model focuses on straightforward, coordinated collaboration by connecting complementary skills, aligning day‐to‐day working practices, and using shared project plans to maintain momentum. BioVentureHub has extended its area of focus to include SustainTech where it, among other areas, explicitly targets green chemistry and biocatalysis expanding the model into a cross‐industry sustainability technology aimed at accelerating development of innovation in these areas [71].

Collectively, these partnerships expand AstraZeneca's biocatalysis capabilities by combining academic innovation with industrial translation, accelerating the development and implementation of new technologies.

## 3 AstraZeneca's Case Studies

Biocatalysis has driven innovation at AstraZeneca since the late 1990s. In the past 15 years, it has progressed from a niche tool to a mature platform and is now routinely used to produce key intermediates for active pharmaceutical ingredients (APIs). On this basis, we present a brief selection of portfolio examples illustrating the impact of biocatalysis in chemical development.

**SCHEME 4:**
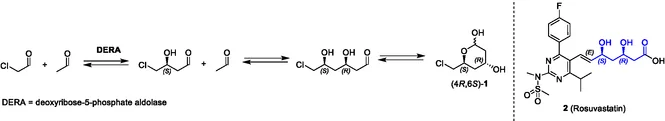
Preparation of chiral lactol (4*R*,6*S*)‐**1** via an engineered deoxyribose‐5‐phosphate aldolase for the synthesis of **2** (Rosuvastatin).

An early prominent example of the value of biocatalysis was the synthesis of the chiral side chain of statins (HMG‐CoA reductase inhibitors), which is essential for activity and often dominates route complexity. Rosuvastatin, marketed as CRESTOR, is widely utilized in the management of hypercholesterolemia and reduction of cardiovascular risk/events. It was approved by the EMA in 2002 and subsequently by the FDA in 2003. Biocatalysis has been demonstrated as a scalable strategy for producing key statin side‐chain intermediates applicable to Rosuvastatin [72, 73, 74]. Enzyme‐based options were evaluated during route development for the synthesis of the chiral lactol (4*R*,6*S*)‐**1** via an engineered deoxyribose‐5‐phosphate aldolase (DERA) from the reaction of chloroacetaldehyde with two equivalents of acetaldehyde en route to **2** (Rosuvastatin) (Scheme 4).

Together with downstream chemical transformations, this biocatalyst‐enabled disconnection provided a practical, scalable statin side‐chain manufacture setting up two stereocenters with 99.9% *ee*. The chemoenzymatic route reduced purification burden by telescoping multistep sequences and, in some cases, avoiding intermediate isolations that would otherwise require solvent‐intensive work‐ups.

**SCHEME 5:**
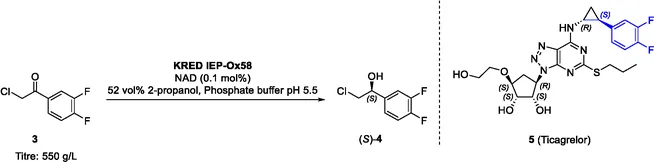
Bioreduction of chloromethyl ketone **3** to synthesize the key chiral alcohol **4** toward **5** (Ticagrelor).

Ticagrelor, marketed as BRILINTA in the US and BRILIQUE in many other markets, provides a particularly instructive example of biocatalysis translated to pharmaceutical manufacture. This orally active antiplatelet drug that blocks the P2Y_12_ subtype of ADP receptors was approved by the EMA in 2010 and the FDA in 2011 for reducing the risk of myocardial infarction and ischemic stroke. Biocatalytic options were evaluated during route development for key intermediates, including work conducted with an external manufacturing partner. The asymmetric reduction of chloromethyl ketone **3** to the key chiral alcohol **4** was performed by a ketone reductase (IEP‐Ox58) under highly concentrated conditions (550 g/L) making the reaction more suitable for industrial scale production toward **5** (Ticagrelor) (Scheme 5).

More broadly, this case highlights the sustainability and performance advantages of enzymatic carbonyl reduction for accessing chiral alcohols [75, 76]: excellent enantioselectivity under mild, aqueous conditions and avoidance of precious‐metal catalysts or borane‐based asymmetric reagents. The demonstration of high substrate loading further underlines the practicality of this approach for large‐scale production.

A sharp increase in the application of transaminases for chiral amine synthesis emerged in the 2000s, driven by the growing availability of commercial enzyme libraries, which enabled broader adoption and more rapid screening, as exemplified by their use in the synthesis of Sitagliptin [77].

**SCHEME 6:**
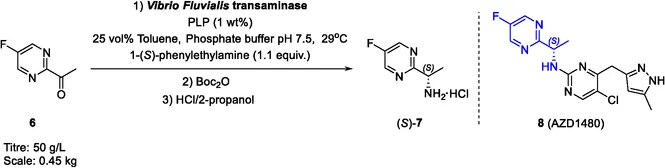
Synthesis of the (*S*)‐amine **7** with *Vibrio fluvialis* transaminase in the synthesis of **8** (AZD1480).

Within this context, a notable success was achieved in the development of the JAK2 kinase inhibitor AZD1480, a small‐molecule candidate developed as a treatment for idiopathic myelofibrosis and polycythemia rubra vera. Evaluation of alternative process options in initial route scouting suggested that biocatalysis had the potential to deliver the required intermediate in a more sustainable manner with high yield and enantioselectivity. Screening of 60 transaminases led to the identification of an enzyme from *Vibrio fluvialis* capable of converting ketone **6** to the (*S*)‐amine **7**. Optimal results were obtained using 1‐(*S*)‐phenylethylamine as the amine donor. The reaction was conducted in a biphasic system of toluene and potassium phosphate aqueous buffer, which allowed efficient extraction of the acetophenone byproduct into the organic layer capable to drove the equilibrium toward product formation. The highly water‐soluble (*S*)‐amine **7** was then isolated by converting it to its Boc derivative and subsequently deprotecting using HCl in isopropanol. Subsequently, the reaction was conducted at 50 g/L on a 0.45 kg scale obtaining (*S*)‐**7** hydrochloride in 77% yield with 99.8% *ee* (Scheme 6) [78, 79].

The transaminase process displayed a sustainability advantage compared to the chemocatalytic alternatives (Ir/Ru/Rh asymmetric hydrogenation or reductive amination) by avoiding costly precious metals and running the reaction in milder aqueous conditions.

As transaminases became increasingly integrated into process development, their utility was exemplified during the development of Savolitinib (AZD6094). Savolitinib is an orally bioavailable, highly selective inhibitor of the MET (HGFR) receptor tyrosine kinase and has demonstrated clinical activity across multiple solid tumors—including lung, renal, and gastric cancers—both as monotherapy and in combination regimens. Early route to Savolitinib relied on reductive amination of ketone **9** with Ellman's chiral sulfoximine [80] to (*S*)‐amine **10**. The overall yield was modest (38% over three stages) and used stoichiometric quantities of titanium ethoxide and diisobutylaluminium hydride. This highlighted an opportunity to deploy biocatalysis to access the corresponding chiral amine under milder, more selective conditions. The initial screening of 106 commercial transaminases revealed seven enzymes that gave >25% conversion to the desired product with (*S*)‐selectivity > 99.5%. A hit transaminase (ATA‐260) was shown to give the desired (*S*)‐amine **10** in >99% conversion using isopropylamine as the amine donor on a small‐scale proof‐of‐concept (0.75 g) albeit at very prolonged reaction time. This promising result initiated an enzyme‐evolution campaign conducted in collaboration with external partners.

A single round of enzyme evolution performed via partners identified ATA‐436 as best enzyme displaying improved performance at pH 10 with >97% conversion to (*S*)‐amine **10** in 72 h. ATA‐436 was chosen for extensive reaction optimization and used successfully to manufacture (*S*)‐amine **10** on a multi‐kilo scale using a 12.5 wt% enzyme. A nitrogen sweep was employed to remove the acetone by‐product to drive the reaction equilibrium toward amine formation. While the above process was successful, enzyme charge of 12.5 wt% was deemed high making the reaction work‐up for the removal of denatured enzyme via centrifugation a slow process. Moreover, the reaction titer (20 g/L) was only moderate.

**SCHEME 7:**
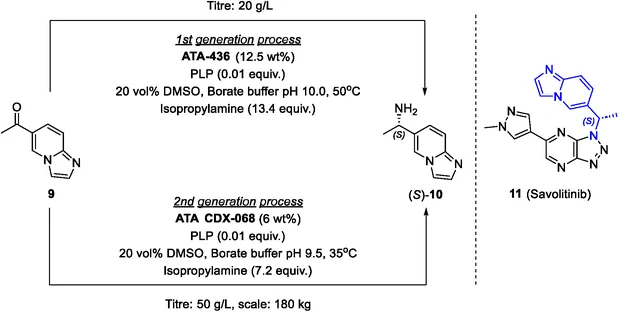
1^st^ generation and 2^nd^ generation biocatalysis process for the preparation of the (*S*)‐amine **10** in the synthesis of **11** (Savolitinib).

In 2019, additional enzyme evolution resulted in the identification of a superior transaminase (CDX‐068). Process scale‐up using CDX‐068 led to a reduction in enzyme charge to 6 wt%, and the substrate concentration increased to 50 g/L. The reaction temperature was lowered to 35 °C, and pH of the reaction mixture was maintained at 9.5 by automated addition of 4 M isopropylamine in water. These changes gave an increased yield of the (*S*)‐amine **10** which was isolated as the dihydrochloride salt from 77 to 88%. The manufacturing process has been carried out on a 180 kg scale (Scheme 7) [81].

Projects such as Savolitinib exemplify a practical development philosophy in industrial biocatalysis: Early enzyme solutions are deployed when they are “fit for purpose” for the immediate delivery and scale and then iteratively improved as requirements tighten for productivity, robustness, and manufacturability in later phases.

**SCHEME 8:**
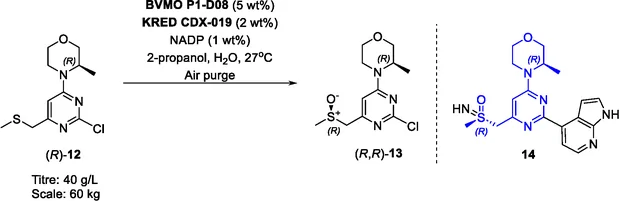
Synthesis of the chiral sulfoxide **13** en route to **14**.

Another significant example was the synthesis of a key chiral intermediate to an ATR Inhibitor. Originally, the chiral fragment was obtained through a multistep route that used *m*‐CPBA oxidation of sulfide **12**, and the resulting diastereomeric mixture of sulfoxides **13** was separated by chromatography. Because this approach could deliver no more than a 50% yield, an alternative strategy was pursued to introduce the sulfoxide functionality in a diastereoselective fashion. However, attempts to apply Kagan‐type conditions [82] resulted in low yields and poor diastereoselectivity. Next, the attention turned to emerging reports describing the asymmetric oxidation of sulfides using Baeyer–Villiger monooxygenases (BVMO), including their successful application in the synthesis of Esomeprazole [83]. To access suitable BVMOs, a collection was purchased from partners and screened. After extensive process development with lead BVMO (P1‐D08), the manufacture was initially performed on a 1.0 kg scale with the BVMO enzyme loading undergoing progressive reduction from 24.0 to 5.0 wt%. With the reduction in BVMO loading, the work‐up was modified to include reduced pressure distillation of 2‐propanol in the reaction mixture followed by 2‐butanone extraction of the sulfoxide. This process was performed at 40 g/L on a 60.0 kg scale, affording (*R*,*R*)‐**13** in 74% isolated yield and > 99% *de*. Successful scale‐up depended on effective aeration of the reaction mixture. To achieve this, air was delivered via a dip pipe submerged in the vessel to ensure sufficient oxygen mass transfer (Scheme 8) [84, 85].

Enzyme implementation was cost‐neutral at kilogram scale, as gains in throughput and reduced raw‐material usage offset enzyme costs. Moreover, the chemoenzymatic route was cost‐saving versus the original unselective route requiring chromatography or crystallization.

At a later stage, the manufacturing route was revisited, and a new cyclopropyl‐substituted sulfide intermediate **15** was introduced into the synthetic sequence to access Ceralasertib (AZD6738). When **15** was subjected to Kagan conditions, the same previous challenges were encountered, affording chiral sulfoxide **16** with modest diastereoselectivity (44% *de*) only. In addition, the lead BVMO P1‐D08, which had performed well in earlier work, delivered both low conversion and poor diastereoselectivity with the new substrate.

To address this limitation, the screening of new BVMO panels was triggered, ultimately identifying a suitable biocatalyst—cyclohexanone monooxygenase from *Rhodococcus ruber* (accession number AAL14233.1)—capable of effecting the desired oxidation. Process development enabled successful scale‐up, and the biotransformation was demonstrated at pilot plant scale using 64.0 kg of sulfide **15**. The reaction was performed with BVMO lysate solution and a 2:1 nitrogen/air mixture purged through the reaction to support biocatalyst turnover. Under these conditions, chiral sulfoxide **16** was obtained in 88% isolated yield with >99% *de*. Moreover, a safe and scalable metal‐free sulfoximine formation was developed, and then optimization of a Suzuki reaction enabled the manufacture of Ceralasertib **17** with excellent control of impurities and an overall yield of 16% [86].

The case of Ceralasertib exemplifies the shift of AstraZeneca's approach to biocatalysis with the integration of internal enzyme evolution capability. Subsequent internal development leveraged computational design, point mutation, and directed evolution to generate a proprietary BVMO enzyme. This enzyme, combined with a complementary KRED recycling enzyme and optimized reaction conditions, enabled reproducible pilot‐scale manufacturing and process validation, underscoring the value of maintaining internal expertise and IP ownership.

**SCHEME 9:**
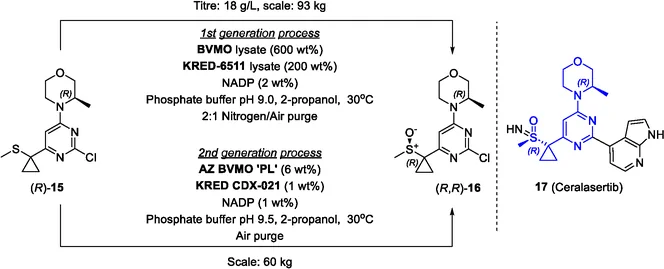
1^st^ generation and 2^nd^ generation process for the synthesis of the key chiral sulfoxide (*R*,*R*)‐**16** en route to **17** (Ceralasertib).

This BVMO enzyme has now been used in two separate pilot‐scale manufacturing campaigns carried out on 60.0 kg scale with a 6.0 wt% loading of the AZ BVMO enzyme, 1.0 wt% of the KRED recycling enzyme CDX‐021, and 1.0 wt% NADP at 30 °C. For the last two campaigns, an air purge of the reaction vessel has been used (the co‐solvent 2‐propanol charge is below the flammable limit) as this was found to enhance conversion (Scheme 9).

The biocatalytic oxidation replaced a low‐yielding, waste‐intensive, and chromatography‐dependent chemical route with a highly selective, metal‐free, and scalable process operating under mild conditions, delivering substantial reductions in waste and improved sustainability.

**SCHEME 10:**
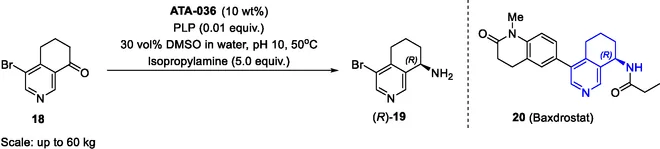
Synthesis of the key chiral amine (*R*)‐**19** en route to **20** (Baxdrostat).

In early 2023, AstraZeneca completed the acquisition of CinCor Pharma, Inc., including Baxdrostat (trade name BAXFENDY), an aldosterone synthase inhibitor being developed for the treatment of hypertension. The synthetic route employed a commercially available transaminase for the transamination of ketone **18** to the chiral amine (*R*)‐**19**. However, the yield for this stage was modest (57%), and the enzyme loading was very high (100 wt% based on input ketone). The modest yield was attributed to product losses during the reaction work‐up (most likely amine product binding to the denatured enzyme removed via filtration). Opportunities were identified to improve the process and triggered the screen of other commercially available transaminases that led to the identification of ATA‐036 as a viable alternative enzyme that could be used with a significantly lower enzyme loading. Indeed, ATA‐036 was used in two consecutive manufacturing campaigns (20.0 and 60.0 kg scale, respectively) applying an enzyme loading of 10 wt% and isopropylamine as the amine donor. The manufacturing conditions utilized periodic distillation of the reaction mixture to remove acetone produced in the process thereby driving the reaction equilibrium toward amine formation. The overall yield in the manufacturing campaigns was 80% after isolation of (*R*)‐**19** as the mono tartrate salt (Scheme 10).

The Baxdrostat program provides a further demonstration of the strategic importance of maintaining a strong internal enzyme‐engineering capability. In parallel with the last manufacturing campaign, an internally driven enzyme‐engineering effort was initiated to eliminate reliance on an externally supplied biocatalyst and to establish a robust, fully in‐house alternative suitable for manufacture. This effort resulted in the identification and implementation of a fit‐for‐purpose transaminase, which was subsequently adopted for the next campaign. Detailed accounts of the enzyme development and associated process optimization will be reported separately.

## 4 Summary and Outlook

The sections above show the breadth and maturity of biocatalysis within AstraZeneca and its increasing role in more sustainable pharmaceutical manufacturing. They also reflect a broader shift across the industry: Biocatalysis is becoming strategically important as companies aim to meet sustainability targets while delivering development programs at pace. In day‐to‐day practice, however, choices are still shaped by speed, robustness, and predictability. This can favor established chemistries when portfolios are under time pressure. For biocatalysis to remain a routinely chosen and industrially competitive option, it must keep improving against these operational requirements.

In response to internal needs and advances in the field, AstraZeneca's biocatalysis capability has evolved into a more integrated function that combines bioinformatics, molecular biology, protein engineering, and process chemistry. Further growth in digital and computational approaches—including machine learning, in silico prediction, and modern data systems—will be important for faster enzyme discovery and more reliable process implementation. The priorities are clear: access a wider range of enzymes; shorten design–build–test cycles; and use large, diverse datasets to support enzyme selection, engineering decisions, and route planning with greater confidence. End‐to‐end automated design–build–test–learn workflows, increasingly linked to machine‐learning‐guided experiment selection, should further improve alignment between enzyme development and industrial timelines. In parallel, extending biocatalytic platforms to large molecules and emerging modalities is expected to broaden the range of reactions that can be addressed.

Together, these advances should improve the predictability of scale‐up and the reliability of technology transfer—two longstanding barriers to wider adoption. Even so, turning new enzymatic transformations into robust, manufacturing steps remains a central challenge. Closing this gap will require continued collaboration across the biocatalysis ecosystem, including strong industry–academia partnerships, to ensure that new chemistries are developed and tested under process‐relevant conditions.

AstraZeneca is committed to continued investment in enzyme engineering, end‐to‐end automation, and strategic external partnerships. These efforts will help embed biocatalysis more systematically into route design and development, so that biocatalytic options are considered early, applied with appropriate confidence, and deliver consistent value across diverse programs. Taken together, these developments position biocatalysis as a key enabler of efficient, resilient, and more sustainable manufacturing routes for the next generation of medicines.

## Conflicts of Interest

The authors declare no conflicts of interest.

## Data Availability

Data sharing not applicable to this article as no data sets were generated or analyzed during the current study.
